# *Trypanosoma cruzi* Survival following Cold Storage: Possible Implications for Tissue Banking

**DOI:** 10.1371/journal.pone.0095398

**Published:** 2014-04-23

**Authors:** Diana L. Martin, Brook Goodhew, Nancy Czaicki, Kawanda Foster, Srijana Rajbhandary, Shawn Hunter, Scott A. Brubaker

**Affiliations:** 1 Centers for Disease Control and Prevention, Atlanta, Georgia, United States of America; 2 University of California, Berkeley, California, United States of America; 3 University of Georgia, Athens, Georgia, United States of America; 4 Center for Medical Technology Policy, Baltimore, Maryland, United States of America; 5 Center for Tissue Innovation and Research, Kettering, Ohio, United States of America; 6 American Association of Tissue Banks, McLean, Virginia, United States of America; French Blood Institute, France

## Abstract

While *Trypanosoma cruzi*, the etiologic agent of Chagas disease, is typically vector-borne, infection can also occur through solid organ transplantation or transfusion of contaminated blood products. The ability of infected human cells, tissues, and cellular and tissue-based products (HCT/Ps) to transmit *T. cruzi* is dependent upon *T. cruzi* surviving the processing and storage conditions to which HCT/Ps are subjected. In the studies reported here, *T. cruzi* trypomastigotes remained infective 24 hours after being spiked into blood and stored at room temperature (N = 20); in 2 of 13 parasite-infected cultures stored 28 days at 4°C; and in samples stored 365 days at −80°C without cryoprotectant (N = 28), despite decreased viability compared to cryopreserved parasites. Detection of viable parasites after multiple freeze/thaws depended upon the duration of frozen storage. The ability of *T. cruzi* to survive long periods of storage at +4 and −80°C suggests that *T. cruzi*-infected tissues stored under these conditions are potentially infectious.

## Introduction

*Trypanosoma cruzi* is a parasite that causes human Chagas disease (American trypanosomiasis). Transmission typically occurs in areas of Latin America where substandard housing provides a habitat for the triatomine bug vectors that deposit the parasite in fecal matter during a nighttime blood meal. The parasite can enter the bloodstream through the skin via any break in the skin or through contact with a mucous membrane. In the vertebrate host, the parasite exists in both intracellular and extracellular forms. Extracellular trypomastigotes can invade virtually any nucleated cell type. Once inside the cell, trypomastigotes transform to amastigotes and replicate [1]. Amastigotes transform back to trypomastigotes after approximately nine rounds of replication over 4–7 days and escape the cell. The released trypomastigotes can be taken up by a triatomine vector during a blood meal or can propagate the infection *in vivo* by infecting other host cells. The acute phase of infection lasts for 1–2 months, during which the parasite has a broad tissue distribution and parasitemia is patent. After the acute phase, parasites persist primarily, but not exclusively, in muscle tissue, and the predominant clinical pathology is cardiomyopathy. Although infection of the host is life-long, parasites are rarely seen in the blood during chronic infection, and even sensitive polymerase chain reaction (PCR) assays only detect parasites in the blood of up to 66% of chronically-infected individuals [2].

To date, 23 cases of vector-borne infection have been identified in the United States (U.S.) [3]. Other potential routes of human transmission in the United States include congenital infection and infection via transplantation and transfusion [4], [5]. An estimated 300,000 *T. cruzi*-infected individuals reside in the U.S. [6]. In the U.S., nine cases of *T. cruzi* transmission from solid organ donation have been documented [5], [7], [8]. Eight reported cases of *T. cruzi* transmission via blood transfusion have been reported; blood donor screening was implemented in the United States in 2007 [9].

The risk of transmission through transplantation of tissues from infected donors is unexplored. Tissue banks oversee the donation of a number of non-solid-organ tissues such as skin, long bone, tendons, ligaments, cornea, heart valves, musculoskeletal tissue, and nerve tissue. Unlike solid organ donation, recovery of tissue may take place up to 15–24 hours after asystole, and many tissues are processed and stored prior to transplantation [10]. While some tissues undergo minimal processing and are stored in cryoprotectant to preserve function (e.g. reproductive tissue and heart valves), other tissues undergo more extensive processing and cold storage in the absence of cryoprotectant prior to transplantation. Some tissue, such as musculoskeletal tissue, can be stored at <−40°C in the absence of cryoprotectant for up to 5 years per American Association of Tissue Bank (AATB) Standards [10]. In early 2009, the U.S. Food and Drug Administration (FDA) issued a draft Guidance for Industry (DHHS/FDA/CBER 2009) that suggested all donors of human cells, tissues, and cellular and tissue-based products (HCT/Ps) be screened and tested for antibodies to *T. cruzi*. This document identified *T. cruzi* as a “relevant communicable disease agent” and described “current data are insufficient to identify specific effective processing methods that consistently render HCT/Ps free of *T. cruzi*.” This announcement coincided with the availability of one FDA-licensed donor screening test for *T. cruzi* antibody that additionally has a claim for use in testing blood specimens from “cadaveric donors of HCT/Ps” [11]. To evaluate this disease agent's relevance to tissue types recovered from deceased donors, studies were undertaken to evaluate the survivability of *T. cruzi* using storage temperatures commonly used for transplantable tissue (i.e. ambient, refrigerated, frozen, and cryopreserved). The aim of this study was to determine the viability of *T. cruzi* parasites following room temperature and cold storage of cell lines infected with *T. cruzi* or of trypomastigotes.

## Materials and Methods

### Human subjects

Blood was collected from a healthy adult volunteer via venous puncture. Written informed consent was obtained under protocol 6062 approved by the New England Institutional Review Board and the Centers for Disease Control and Prevention Human Subjects Office.

### Cell lines and parasite strains

Three human cell lines and one monkey-derived cell line were used in this study (HMEC-1, a human microvascular endothelial cell line; FS9, a human foreskin fibroblast cell line; Chang CONJ, a conjunctival epithelial cell line, and Vero, a green monkey kidney epithelial cell line). All cell lines were acquired from the Division of Scientific Resources at CDC. Three strains of *T. cruzi* were used (Brazil, Y, and Tc23). Brazil and Y are both long-standing laboratory strains; Tc23 was isolated from a guinea pig in the Arequipa district of Peru in 2009, passaged in cell culture, and maintained as frozen stocks (Martin, et. al, *AJHTM*, in press).

### Cell infection and culture

All cells were cultured in tissue-culture treated flasks in a humidified, CO_2_-rich, 37°C environment using RPMI-10 (Life Technologies, Grand Island, NY) media supplemented with 10% fetal bovine serum (FBS, Fisher Scientific, Pittsburgh PA), L-glutamine (Life Technologies), sodium pyruvate (Life Technologies), and penicillin/streptomycin (Life Technologies) (hereafter referred to as RPMI-10). All cell lines were adherent and removed from the flask for storage by incubation with 0.25% trypsin in EDTA (LifeTechnologies) for 1–3 minutes, followed by washing in RPMI-10. In this study, trypomastigotes from all parasite strains were able to infect all cell lines tested. During culture for viability testing, if cells became confluent, they were trypsinized and split into additional flasks for further culture.

### Cell recovery and storage

Parasites and parasite-infected cells were stored at three temperature ranges (room temperature, refrigerated, and frozen) and various lengths of time for determination of parasite viability following storage. For room temperature storage (22–25°C), 1 ml of venous blood from a healthy, uninfected adult volunteer was spiked with 2×10^6^ trypomastigotes. In some trials blood was collected in heparin, in other trials the blood was allowed to clot. Spiked blood was stored at room temperature for 24 hours, after which blood was examined on a slide for motile trypomastigotes and then plated on uninfected cells for assessment of parasite infectivity. To represent refrigerated temperature storage (from above 0°C to 10°C), cells from an infected flask were released by trypsinization and aliquoted into 15 ml conical tubes, or infected flasks themselves were placed in a refrigerator for periods of 24 h, 48 h, 72 h, 5 d, 7 d, 10 d, 14 d and 28 d. For frozen (−80°C) storage, cells from an infected flask were trypsinized and resuspended in a cryoprotectant solution (90% fetal bovine sera (FBS)/10% dimethylsulfoxide (DMSO, Sigma-Aldrich, St. Louis, MO) as a positive control, 100% FBS, or RPMI -10. In other experiments, trypomastigotes were removed from the culture supernatant, washed, and stored in cryoprotectant solution, FBS, or RPMI-10 at 1×10^6^ or 5×10^6^ parasites per sample. Trypomastigotes or infected cells were aliquoted to cryogenic tubes and placed in a Mr. Frosty freezing container (Nalge Nunc, Penfield, NY, USA), to achieve a freezing rate of −1°C/minute in a −80°C freezer, and then were stored in a −80°C freezer for 24 h, 48 h, 72 h, 5 d, 14 d, 30 d, 60 d, 90 d, 120 d, and >365 days. The work flow is outlined in Table 1.

### Freeze-thaw analysis

Trypomastigotes were frozen as described in RPMI-10 with or without cryoprotectant. Samples were thawed, washed once in RPMI-10, and then refrozen in the same condition (i.e. RPMI-10 alone or with cryoprotectant) as the first freeze. This was repeated for a total of 4 freeze-thaw cycles with an interval of either 1 week or >30 days between each freeze/thaw.

### Measures of cell viability

**Figure 1:**
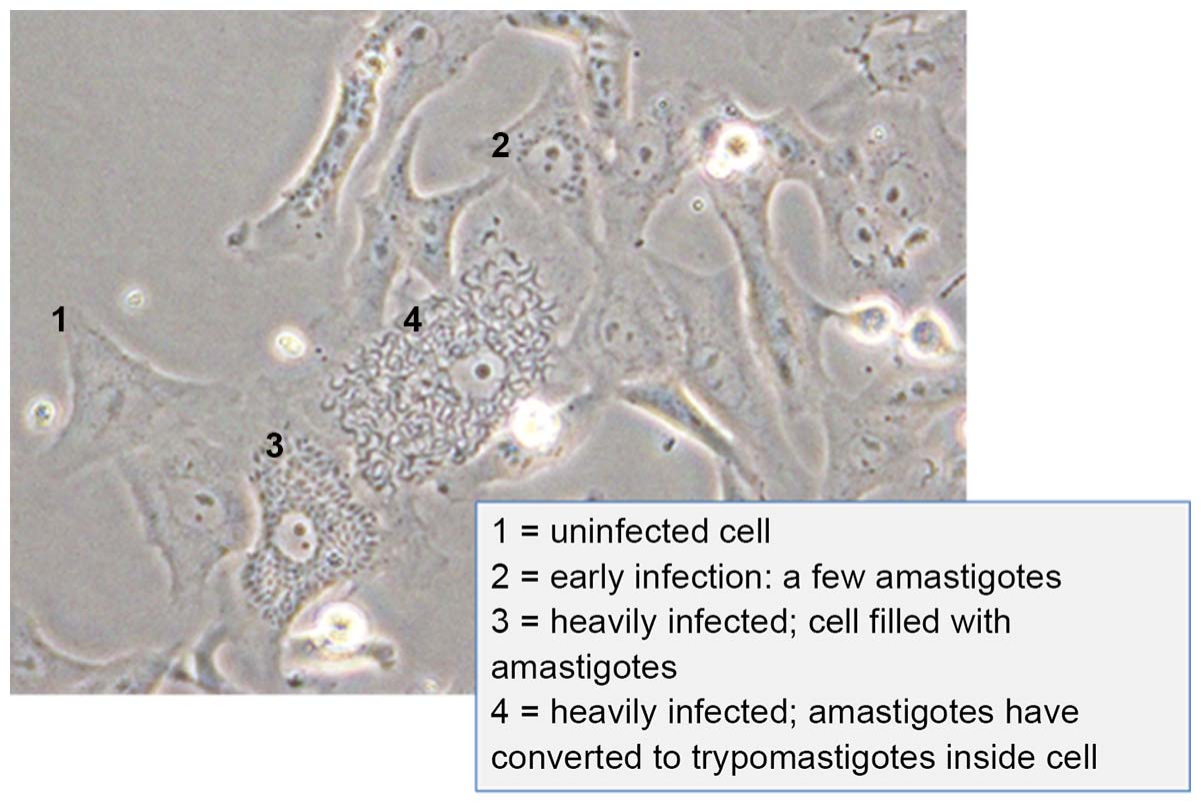
Vero cell culture infected with Brazil strain *T. cruzi* showing asynchronous amastigote nests inside cell. (1) uninfected cell; (2) very early infection, (3) more advanced and (4) a cell in which amastigotes have completely transformed to trypomastigotes. Note that other infected cells are present in this view as well. Photo was taken at 400× magnification.

In all studies, samples were cultured over uninfected mammalian cell lines at 37°C in a humidified 5% CO_2_ incubator for 7 days to 3 months. Cultures were examined under 200× and 400× for the presence of motile trypomastigotes in the culture supernatant and the presence of amastigote nests in cells (see Figure 1). Either of these were indicators of viable parasites. Parasites spiked into blood for 24 h room temperature storage were examined on a slide (10 µl) for live parasites prior to re-culture. To compare the number of viable parasites stored at −80°C in cryoprotectant solution, FBS alone, or RPMI-10 alone, parasites were thawed, washed once in RPMI-10, and resuspended in 1 ml of RPMI-10 before being enumerated on a hemocytometer. All samples were then added to fresh mammalian cells to determine parasite infectivity as described above.

### Photos and videos

Digital photos and videos were taken at 400× magnification or 200× magnification as listed in figure legends using an Olympus DP21 camera (Tokyo, Japan) connected to an Olympus CKX41 microscope. Videos were filmed at 15 frames per second and saved as .AVI files.

## Results

### Effect of room temperature storage on *T. cruzi* viability in the presence of decaying blood

**Figure 2:**
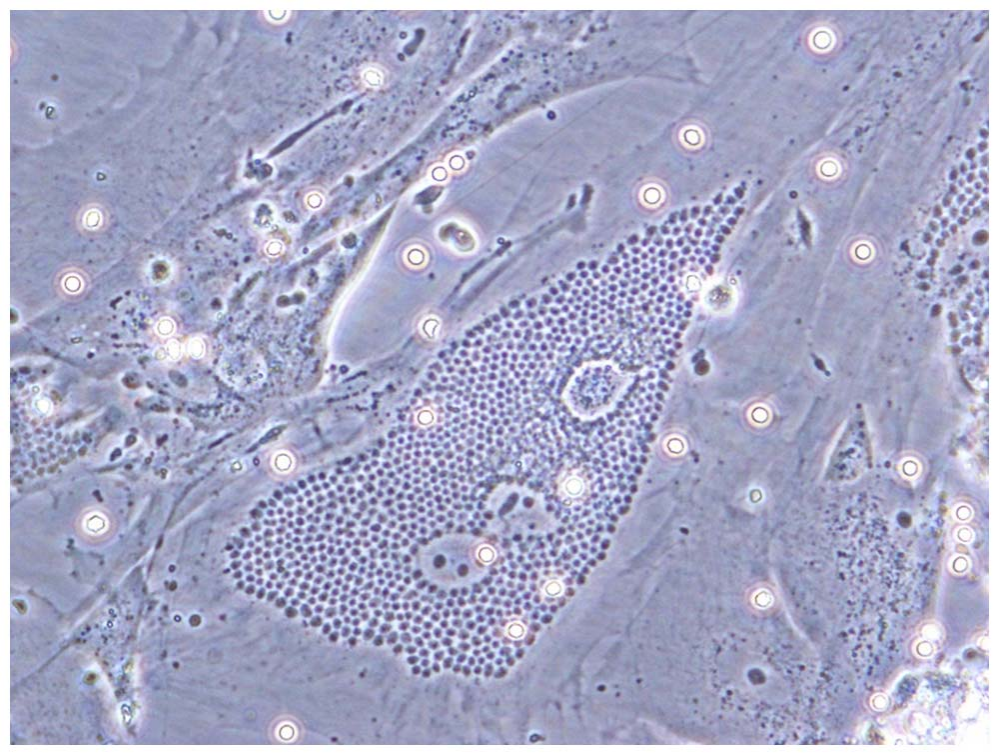
Infectivity of parasites stored 24 hours at RT in decaying blood product. One ml of heparinized (n = 5) or non-heparinized (n = 7) blood was spiked with 2×10^6^ Y or Tc23 *T. cruzi* trypomastigotes. Blood was cultured over FS9 or HMEC-1 cells and examined for amastigote nests after 4–7 days. Pictured is a representative amastigote nest of Y strain parasites in FS9 cells. Photos were taken at 400× magnification.

Motile parasites were observed in *T. cruzi*-spiked blood under a light microscope following 24 hours of room temperature storage (Video S1). Infected cells were observed following plating with the spiked, stored blood (Figure 2). Motile, infective parasites were present in both heparinized (n = 8) and coagulated (n = 5) spiked blood samples.

### Effect of refrigerated temperature storage on *T. cruzi* viability

**Figure 3:**
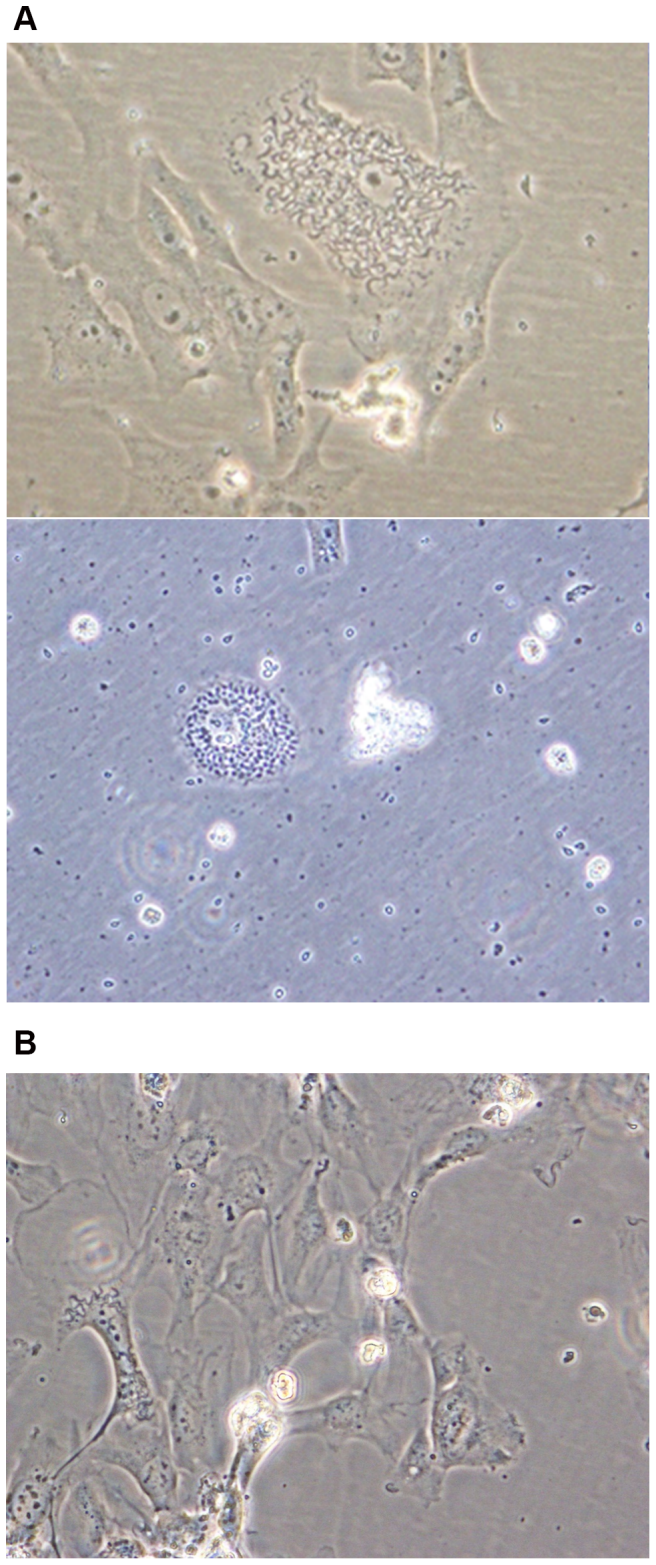
*T. cruzi*-infected culture cells were stored for various lengths of time at 4°C. A. Vero cells infected with Brazil *T. cruzi* after 24 h at 4°C, then re-cultured at 37°C. Figure is representative of N = 7. B. HMEC-1 cells infected with Tc23 *T. cruzi* were stored 5 d at 4°C, then the supernatants were re-cultured over fresh HMEC-1 cells. Figure is representative of N = 10 stored cultures and positive re-cultures.

*T. cruzi*-infected cells stored at refrigerated temperature for 24 h and 48 h exhibited no change in cell or parasite viability upon re-culture (Figure 3A) but showed a qualitative decrease in viable cells but not parasites when stored 72–120 hours at refrigerated temperatures (data not shown and Figure 3A). When stored between 5 and 10 days at 4°C, re-culture of supernatants over uninfected mammalian cells showed infective trypomastigotes (Figure 3B and data not shown). Following 14 days at refrigerated storage temperatures, no swimming parasites were seen in the stored flasks and no culture-positive flasks were observed after re-culture (n = 9). The majority (11/13) of *T. cruzi*-infected flasks stored 28 days at refrigerated temperatures had no viable parasites upon re-culture, but two samples showed infective parasites upon culture with uninfected cells, although these were not visible until one month later (Video S2).

### Effect of frozen storage on *T. cruzi* viability

**Figure 4:**
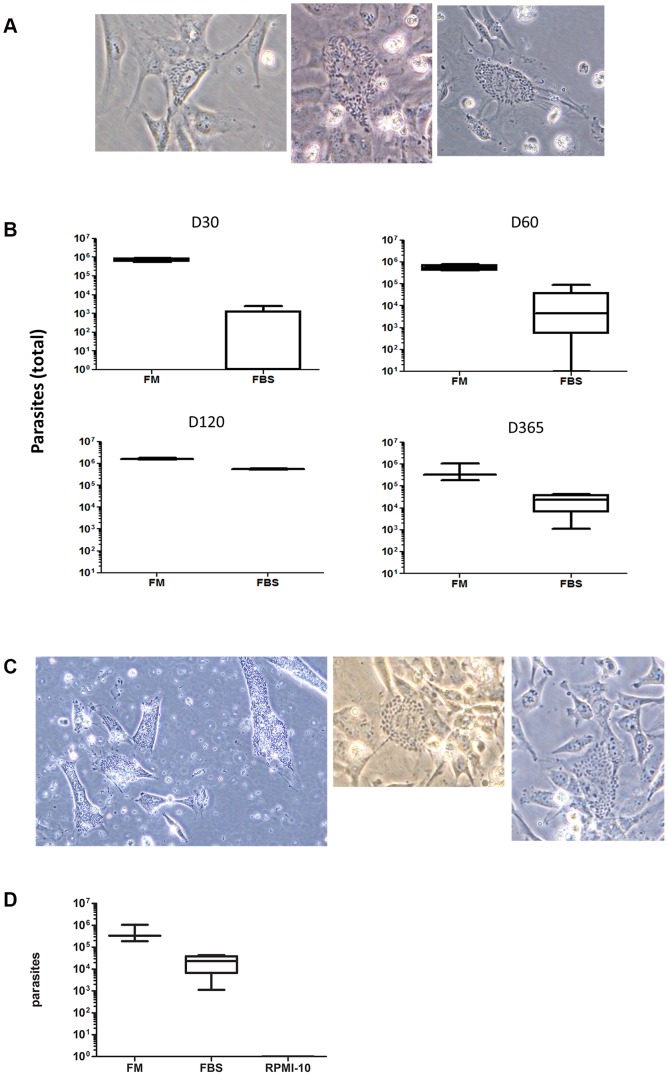
Viable parasites recovered after one year storage at −80°C without cryoprotection. A. *T. cruzi* (Tc23 stain)-infected HMEC-1 cells were stored in RPMI-10 at −80°C for greater than one year, then re-cultured in HMEC-1 cells. Amastigote nests were photographed at 400× magnification. Data are representative of N = 19 cultures. B. *T. cruzi* trypomastigotes (Tc23 strain) were stored with cryoprotectant (freezing media, FM) or FBS at −80°C for the indicated time period. Samples were thawed, washed in RPMI-10, and counted on a hemocytometer. Data represent at least N = 4 per group. C. *T. cruzi* trypomastigotes (Tc23 strain) were stored in RPMI-10 at -80°C for greater than one year, and then re-cultured in HMEC-1 cells. Infected cells were photographed at 400×. Data are representative of N = 5 cultures. D. *T. cruzi* trypomastigotes (Tc23 strain) were stored in FM, FBS, or RPMI-10 at −80°C for greater than one year. Samples were thawed, washed in RPMI-10, and counted on a hemocytometer. Data represent N = 4 per group.

Frozen storage of *T. cruzi*-infected cells in the absence of cryoprotectant resulted in low cell and parasite viability within 24 h, but a small number of viable parasites were still observed. Viable parasites capable of infecting cells in culture were present at one year at −80°C frozen storage even in the absence of cryoprotectant (Figure 4A).

*T. cruzi* trypomastigotes were enumerated on a hemocytometer after storage at −80°C in the presence or absence of cryoprotectant for varying lengths of time. The number of viable parasites recovered after 30, 60, 120, and 365 days of storage from those stored with cryoprotectant was significantly higher than in parasites stored without cryoprotectant, although viable parasites stored in in the absence of cryoprotectant were still observed on a hemocytometer following as long as 6 months of frozen storage (Fig 4B). After one year of frozen storage in RPMI-10, parasites could no longer be enumerated on a hemocytometer directly after thawing, but were still able to infect cell cultures (Fig 4C). Storage in 100% FBS yielded higher recovery of viable parasites, as parasites were observed on a hemocytometer after one year of frozen storage in FBS (Fig 4D). In all conditions, plating the samples from frozen storage on HMEC-1 or FS9 cells resulted in infected cultures 3 to 5 weeks later (Fig 4C).

Parasites subjected to four freeze/thaw cycles with storage in either culture media or cryoprotectant at one-week intervals were viable (Video S3). However, when the storage time between freeze/thaws was increased to greater than one month, no viable parasites or positive re-cultures were observed (data not shown).

## Discussion

Because *T. cruzi* exists as both intracellular and extracellular forms, the potential for transmission from cellular and acellular tissue grafts and blood products exists. Solid organ donation of the heart [5], liver [8], kidney and kidney/pancreas [8], [12]–[16] combination, lungs [7], bone marrow [17], [18], and cord blood [19] from infected donors has resulted in transmission to recipients in both endemic and non-endemic countries. Tissue donation presents a unique scenario due to the possibility of parasites being exposed to decaying blood and tissue in the deceased donor prior to tissue recovery, undergoing chemical processing, irradiation, or lyophilization during tissue processing, and being placed in short-term storage at refrigerated temperatures and long-term storage at frozen temperatures. In this study, we addressed the effects of exposure to products from decaying blood, short-term refrigerated storage, and long-term frozen storage on *T. cruzi* parasites. The data presented herein show that *T. cruzi* is able to withstand all of these conditions, although cold storage does significantly decrease the number of viable parasites present.

Many types of tissue for transplant may be recovered from a deceased donor up to 15 hours after asystole, during which time the donor's body may not yet be stored at refrigerated temperatures [10]. Parasites present within the tissue would therefore be exposed to products from decaying blood which might impact the parasite viability. We sought to mimic this by spiking trypomastigotes into freshly-obtained venous blood and letting the parasites remain within the blood as it decayed. Viable trypomastigotes were observed by microscopic examination in either heparinized or non-heparinized blood after the 24-hour storage period, and these readily infected cell cultures.

While the vast majority of parasites die during long-term cold storage, the presence of small numbers of viable, infective parasites suggest a low potential for transmission of *T. cruzi* from stored tissue. In refrigerated and frozen conditions, the parasites showed greater resistance to death than the mammalian culture cells used. Parasites appeared to be more susceptible to refrigerated storage than frozen storage, although this was a qualitative and not quantitative observation. It is not clear from the current studies whether parasite death in refrigerated conditions is an active process induced by toxic products released from the dead culture cells or simply due to starvation. Other reports show *T. cruzi* survival following 18 days in refrigeration [20], [21], so our study may underestimate the number of infective parasites following 14 days of refrigerated storage. In the absence of cryoprotectant, *T. cruzi* trypomastigotes fared better when frozen in 100% serum than in media containing 10% serum. It may therefore be important to consider the water content of tissue when thinking about *T. cruzi* survival during storage.

Tissue stored for transplantation can be thawed for testing and re-frozen. We therefore examined the viability of *T. cruzi* following up to 4 freeze-thaws. When the length of storage between freeze-thaws was short (one week), infective parasites were obtained in all cultures, but when the length of storage was extended to one month between freeze-thaws, no viable parasites were observed. While parasites were highly susceptible to the combined stress of long-term frozen storage and multiple freeze-thaws, not all tissues are routinely subjected to these conditions to ensure the destruction of *T. cruzi* parasites.

*T. cruzi* may not persist in all tissue in chronically-infected individuals, so knowing which tissues are affected in chronically infected individuals would be useful in future decision-making about transplantation of tissue from infected donors. The tissue distribution of *T. cruzi* in acute experimental infection of mice is extensive and involves virtually all tissues [22]–[26], but the extent to which *T. cruzi* persists in different tissue during chronic infection is poorly understood. A prospective study of 9 liver grafts transplanted from *T. cruzi*-infected donors to seronegative patients in Argentina showed only 2 parasitemia-positive outcomes in a one-year follow-up [27].

Some of the outcomes were difficult to quantitate due to the low sensitivity of the hemocytometer. At least 100 events need to be counted for accurate measurements; however, in many instances only one or two parasites were counted on the hemocytometer following frozen storage in RPMI-10. These data therefore give an inaccurate count, but still demonstrate the presence of motile parasites immediately after thawing. For many specimens, samples in which no parasites were seen on the hemocytometer resulted in infected cell cultures. Because we could not accurately quantify the number of viable parasites in many instances, we instead focused on the qualitative data showing live parasites following culture. Similarly, quantitation was only achievable in conditions in which a positive control group was available, such as frozen storage conditions in which cryopreservation is a well-established technique [28]. There is no analogous preservation method for *T. cruzi* parasites at refrigerated temperatures.

Cell cultures were used in this study in lieu of animal models primarily due to the failure of positive controls in pilot studies using mice; when muscle tissue from acutely-infected mice was cultured immediately after dissection, only 30% of samples (6/20) yielded positive parasite cultures. In order to show a decrease from a no storage positivity rate of 0.30 to 0.10 for cold storage conditions using paired samples with a McNemar test and Connor approximation [29], an N of 86 per condition would be required (Ryan Wiegand, personal communication), and for unpaired samples using Fisher's exact test [30], an sample size of 180 mice per condition would be required (power analyses were performed in SAS version 9.3, SAS Institute, Inc., Cary, NC). With 72 conditions to be considered for pre- and post-recovery storage, this sample size (6192 mice) made the use of animals not feasible. The use of cell cultures leaves some questions unanswered, such as the role of the immune response in protecting against parasite infection in recipient and the potential protective effect of a large tissue mass on parasites in that tissue.

Other common preservation methods applied to tissues for transplantation, such as lyophilization, air-drying, and various chemical devitalization treatments, and mechanical agitation, are not addressed herein and are the subjects of ongoing studies. Many allograft types may be provided as acellular or decellularized after these common treatments are applied. The reported rate of confirmed *T. cruzi* infection in volunteer U.S. blood donors is approximately 1∶27,500 [9], so with about 30,000 tissue donors annually in the United States, tissue banks should expect to encounter one or two *T. cruzi* infected tissue donors each year. To date, no tissue donation-associated *T. cruzi* infections have been identified. However, two of the transfusion-associated cases in the U.S. received platelet products that had been documented to be leukoreduced and irradiated [9], further showing the high resistance of *T. cruzi* to techniques commonly applied to blood products and tissue allografts. Understanding how other tissue processing techniques affect the viability of the parasite will be critical in light of the data presented here showing that viable, infective parasites remain following long-term cold storage of *T. cruzi*.

## Supporting Information

Video S1 Infectivity of parasites stored 24 hours at RT in decaying blood product. One ml of heparinized (n = 5) or non-heparinized (n = 7) blood was spiked with 2×10^6^ Y or Tc23 *T. cruzi* trypomastigotes. After 24 h at room temperature, blood was examined for viable trypomastigotes. Video shows swimming trypomastigote amongst red blood cells.
(AVI)

Video S2 Trypomastigotes in culture after 28 d storage at 4°C. Video is representative of 1 positive culture out of 13 total tested (1 additional positive sample was not re-cultured due to extensive yeast contamination). Video was taken at 400× magnification.
(AVI)

Video S3 Effect of multiple freeze/thaws on parasite viability in the absence of cryoprotection. Trypomastigotes were stored in RPMI-10 at −80°C for one week, between four total freeze/thaw cycles then re-cultured in HMEC-1 cells. Video was taken at a 200× magnification. Data are representative of N = 5 cultures.
(AVI)
