# Correction: *Trypanosoma cruzi* Survival following Cold Storage: Possible Implications for Tissue Banking

**DOI:** 10.1371/journal.pone.0114783

**Published:** 2014-12-03

**Authors:** The PLOS ONE Staff

Table 1 does not appear in the published article. Please view Table 1 here as Figure S1 of this correction.

## Supporting Information

Figure S1 **Storage conditions.**
(DOCX)
