# Supplementary figures and images for: Correction: Trypanosoma cruzi Survival following Cold Storage: Possible Implications for Tissue Banking
Source: PLoS One. 2014 Dec 3;9(12):e114783. doi: 10.1371/journal.pone.0114783 (PMC4255030; doi:10.1371/journal.pone.0114783)

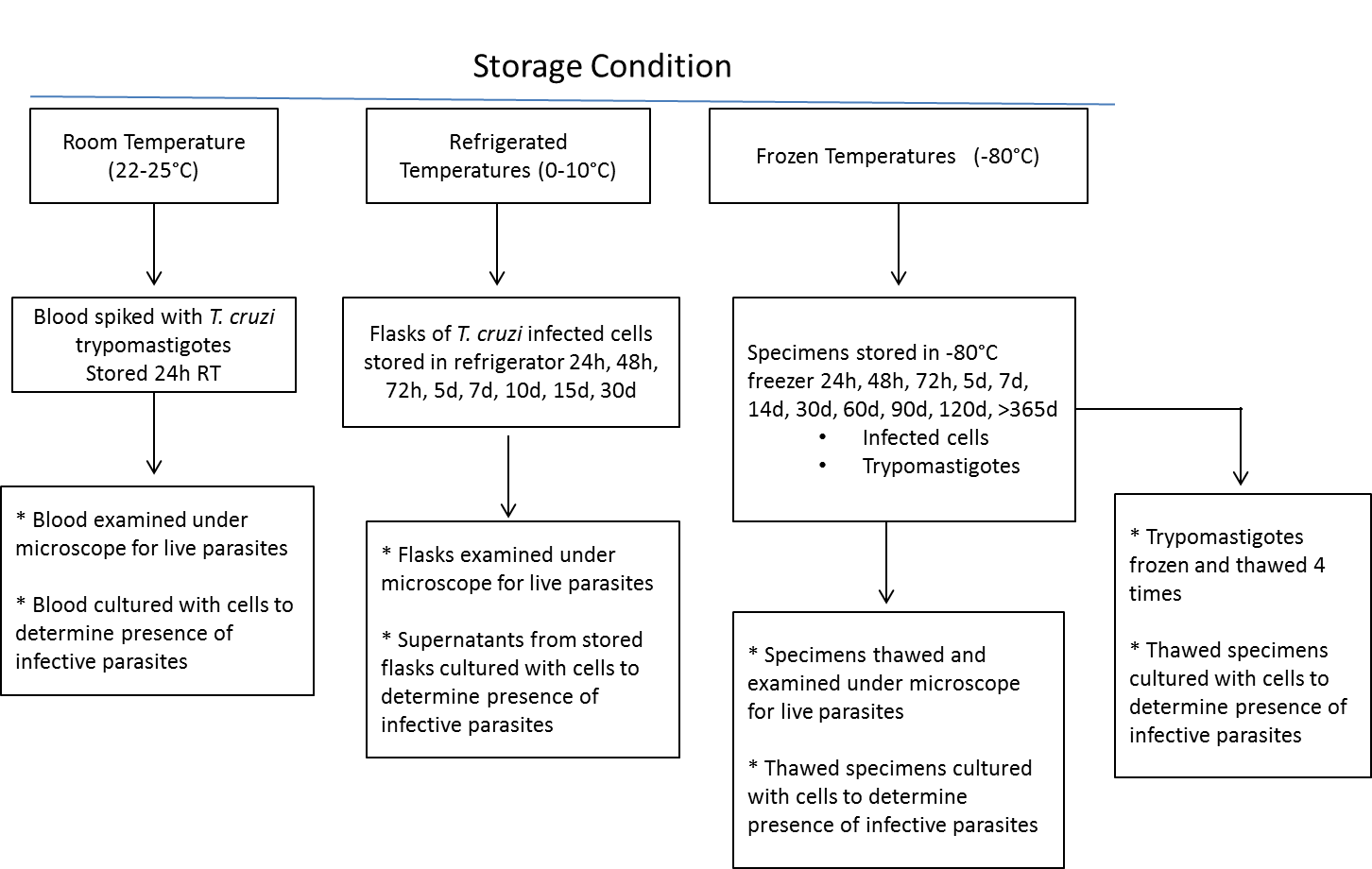

Supplement: Figure S1 — Storage conditions. (DOCX) [file pone.0114783.s001.docx]
